# Rumination in major depressive disorder is associated with impaired neural activation during conflict monitoring

**DOI:** 10.3389/fnhum.2015.00269

**Published:** 2015-05-12

**Authors:** Brandon L. Alderman, Ryan L. Olson, Marsha E. Bates, Edward A. Selby, Jennifer F. Buckman, Christopher J. Brush, Emily A. Panza, Amy Kranzler, David Eddie, Tracey J. Shors

**Affiliations:** ^1^Department of Exercise Science and Sport Studies, Rutgers, The State University of New JerseyNew Brunswick, NJ, USA; ^2^Department of Nutritional Sciences, Rutgers, The State University of New JerseyNew Brunswick, NJ, USA; ^3^Center of Alcohol Studies, Rutgers, The State University of New JerseyNew Brunswick, NJ, USA; ^4^Department of Psychology, Rutgers, The State University of New JerseyNew Brunswick, NJ, USA; ^5^Department of Psychology, Center for Collaborative Neuroscience, Rutgers, The State University of New JerseyNew Brunswick, NJ, USA

**Keywords:** depression, cognitive control, anterior cingulate cortex, dorsolateral prefrontal cortex, N2, P3

## Abstract

Individuals with major depressive disorder (MDD) often ruminate about past experiences, especially those with negative content. These repetitive thoughts may interfere with cognitive processes related to attention and conflict monitoring. However, the temporal nature of these processes as reflected in event-related potentials (ERPs) has not been well-described. We examined behavioral and ERP indices of conflict monitoring during a modified flanker task and the allocation of attention during an attentional blink (AB) task in 33 individuals with MDD and 36 healthy controls, and whether their behavioral performance and ERPs varied with level of rumination. N2 amplitude elicited by the flanker task was significantly reduced in participants with MDD compared to healthy controls. Level of self-reported rumination was also correlated with N2 amplitude. In contrast, P3 amplitude during the AB task was not significantly different between groups, nor was it correlated with rumination. No significant differences were found in behavioral task performance measures between groups or by rumination levels. These findings suggest that rumination in MDD is associated with select deficits in cognitive control, particularly related to conflict monitoring.

## Introduction

Major depressive disorder (MDD) is one of the most common mental health disorders in the United States with a lifetime prevalence of ∼16.6% (Kessler et al., 2005). Globally, more than 350 million people suffer from depression, and by 2020 it is predicted to be the second largest cause of disability, for all ages and both sexes (Mathers et al., 2008). MDD is characterized by a number of behavioral, emotional, and cognitive symptoms, including psychomotor agitation or retardation, insomnia or hypersomnia, decreased or increased appetite, fatigue, feelings of guilt and worthlessness, and suicidal ideation (American Psychiatric Association, 2000). Other hallmark symptoms include rumination, wherein individuals retrieve and repetitively rehearse autobiographical and negatively valenced content about past and current problems, and attentional problems associated with an inability to focus, concentrate, or sustain attention (American Psychiatric Association, 2000; Davidson et al., 2002). These latter symptoms are indicative of deficits in cognitive functioning, which may further contribute to disability and poor quality of life (Holmes and Pizzagalli, 2008; Hammar and Ardal, 2009; Gotlib and Joormann, 2010).

Rumination is one of the most problematic cognitive symptoms associated with depression. These negative thought processes heighten negative affect and interfere with an individual’s ability to engage in effective problem-solving and adaptive behaviors (Nolen-Hoeksema et al., 1999; McLaughlin et al., 2007; Nolen-Hoeksema et al., 2008). A number of studies suggest that MDD is associated with impairments in cognitive control processes, specifically those involved in regulating conflict (Lemelin et al., 1997; Davidson et al., 2002; Vanderhasselt et al., 2012; Clawson et al., 2013). In general, cognitive control reflects a person’s ability to flexibly and voluntarily regulate behavior or thoughts in the service of goal-directed and purposive behaviors while resisting the retrieval and distraction of competing undesirable information (Miller, 2000; Miller and Cohen, 2001; Aron, 2007). Although less well-studied, recent studies also suggest that excessive rumination, as is often found in individuals with MDD, is associated with less cognitive control (Joormann et al., 2006; Whitmer and Banich, 2007). These control processes are involved in many aspects of healthy cognition and may be involved in delay of gratification and impulse control, as well as self-reflection and the intrusion of negative thought patterns. Structural and functional neuroimaging studies implicate prefrontal and anterior cingulate brain regions in cognitive control (Davidson et al., 2002; van Veen and Carter, 2002; Wagner et al., 2006; Holmes and Pizzagalli, 2008). However, the temporal dynamics of conflict monitoring and cognitive control are not well-described, especially as they relate to rumination.

According to the conflict monitoring hypothesis (Botvinick et al., 2001, 2004; Yeung et al., 2004), an essential aspect of cognitive control involves conflict monitoring and conflict detection, both of which are believed to involve important connections between the anterior cingulate cortex (ACC) and lateral prefrontal regions. Several laboratory-based assessments including the go/no-go, stop signal, antisaccade, Stroop, and flanker interference tasks have been used to manipulate and assess conflict monitoring and cognitive control. The flanker interference task represents a canonical example of this response conflict, such that the presence of competing responses associated with the incongruent condition results in impaired performance relative to the congruent task conditions. Successful performance on this task, particularly on the more challenging trials where the flanking arrows are incongruent with the target arrow, requires greater top–down cognitive control. That is, the incongruent task condition requires competition at the level of response activation and a person’s ability to suppress inappropriate or prepotent responses. Task performance deficits have been reported in a number of clinical populations in which disturbances in conflict and response monitoring are present, such as schizophrenia, depression, and substance use disorders (van Veen and Carter, 2002; Pizzagalli, 2011). Functional magnetic resonance imaging (fMRI) and event-related brain potential (ERP) studies suggest a critical role of the ACC in detecting and evaluating conflicts as they emerge during the action selection process, and using this information to signal for increased recruitment of cognitive control from lateral PFC regions (Botvinick et al., 1999, 2001; van Veen and Carter, 2002; Yeung et al., 2004). The N2 ERP component has been instrumental in studying ACC-mediated conflict monitoring in cognitive control and has also been used to study frontocingulate dysfunction in depression. The conflict N2 (sometimes referred to as flanker N2) is a negative deflection in the stimulus-locked ERP with a frontocentral scalp distribution that peaks ∼200–350 ms after stimulus presentation (Botvinick et al., 2004; Folstein and Van Petten, 2008; Clawson et al., 2013). As an index of conflict processing, this ERP component has been shown to be more negative (i.e., larger) for incongruent flanker trials as a result of conflict that arises during response selection between the responses queued by the target stimulus and those queued by the incompatible flanking stimuli (Yeung et al., 2004; Clawson et al., 2013). This response conflict can also be measured behaviorally, and it has consistently been shown that incongruent or conflicting task conditions result in impaired accuracy and increased reaction time relative to congruent task conditions (Yeung et al., 2004; Tillman and Wiens, 2011; Larson et al., 2014).

Previous research examining conflict monitoring processes and the N2 in MDD has been mixed (Kaiser et al., 2003; Bruder et al., 2012). For instance, Ruchsow et al. (2008) examined N2 and P3 ERP components elicited by a hybrid flanker go/no-go task where participants responded to the appearance of letters B or U as centrally located flanker stimuli (“go” condition) and withheld a response to the appearance of letters D or V. Although individuals with MDD evidenced reduced (“less positive”) no-go P3 amplitudes compared to matched healthy controls, no between group differences were noted for the N2 component. Similarly, no between-group differences in response time, error rate, or N2 indices of conflict adaptation were found between 55 individuals diagnosed with MDD and demographically similar control participants using a modified flanker task (Clawson et al., 2013). Although no between group differences were found in conflict adaptation, a cognitive control process involving the influence of previous trial congruency on current-trial performance, higher depressive symptom scores based on the BDI-II were associated with smaller mean N2 conflict adaptation scores for individuals with MDD, suggesting that N2 conflict adaptation may be associated with depressive symptoms rather than a clinical diagnosis *per se*. Alternatively, using an auditory go/no-go task, Kaiser et al. (2003) reported a reduction of inferior frontotemporal positivity in the N2 latency range (i.e., polarity-inverted N2) among patients with unipolar depression. This was interpreted to reflect a specific deficit in the response inhibition component of executive control, and was accompanied by impaired behavioral task performance during the no-go task condition. Differences in the specific tasks used, clinical characteristics of MDD participants, or medication status may have contributed to the mixed findings in the literature. However, the relation between N2 amplitude indices of conflict monitoring and cognitive control and maladaptive rumination remains to be studied.

In addition to deficits in conflict monitoring, individuals with MDD often experience a selective loss of attention and/or attentional inflexibility (Hammar and Ardal, 2009; Lyche et al., 2011), which may impair their ability to multitask, maintain conversations, and ignore distractions. These problems, in turn, often lead to impaired focus and forgetfulness (Ravnkilde et al., 2002). Several studies have demonstrated attentional deficits in individuals with MDD using a variety of attention-related neuropsychological measures (Cohen et al., 2001; Hammar et al., 2003; Lyche et al., 2011). However, there is some inconsistency across studies. Ottowitz et al. (2002) published a review suggesting that the evidence for selective deficits in attention in MDD was equivocal. Indeed, less than half of the studies (44%) included in the review demonstrated attentional impairments in MDD. The lack of consistent findings may have been due to variability in study designs, subtyping of depression (e.g., melancholic versus atypical depression), or may simply reflect the multifaceted nature of attention. That is, MDD may impair only select aspects of attention, and these impairments may be best characterized by tests specifically designed to evaluate a particular component of attention (Ottowitz et al., 2002). Further, rumination may contribute to many of the cognitive biases and impairments found in MDD, including deficits found in attentional processes (Donaldson et al., 2007), but this suggestion has received only limited research attention.

Attentional processes in the brain depend on finely timed sequences that result in the allocation of attentional resources for perception and processing of sensory stimuli across time. One approach that has been used to probe the temporal dynamics of attention is the rapid serial visual presentation (RSVP) paradigm and the attentional blink (AB) phenomenon (Raymond et al., 1992). First described in 1992, the AB is typically observed during RSVP tasks whereby individuals exhibit a reduced ability to report the second (T2) of two different target stimuli presented among a very rapid stream of visual distractors when T2 appears within ∼100–500 ms of the first target (T1; Broadbent and Broadbent, 1987; Raymond et al., 1992). Although no current theoretical explanations fully account for this phenomenon (Dux and Marois, 2009), most point to a limited capacity attentional resource system, indicating that sensory information is not transferred efficiently from early sensory processing stages (and brain regions) to those involved in working memory (Luck et al., 1996; Martens et al., 2002). The magnitude of the AB (i.e., the time it takes to recognize T2 following presentation of T1) has been shown to be larger in clinical populations (Husain et al., 1997; Husain and Rorden, 2003), the elderly (Lahar et al., 2001; Maciokas and Crognale, 2003), and children with attention-deficit/hyperactivity disorder (Hollingsworth et al., 2001; Li et al., 2004). Neuroimaging evidence suggests an interactive neural network consisting predominantly of overlapping lateral-frontal, inferotemporal, posterior-parietal, and occipital brain regions underlying the AB response (Marois and Ivanoff, 2005; Hommel et al., 2006).

For the AB paradigm, we assessed the classic cognitive P3 ERP component, which is thought to reflect the allocation of attentional resources during the updating of working memory (Donchin and Coles, 1988; Polich, 2007). Previous studies have demonstrated a completely suppressed P3 but no change in amplitude or latency for earlier ERP components (e.g., P1, N1, N400), when the AB phenomenon occurs. These findings suggest that the AB occurs after early perceptual processing is complete. It also has been speculated that the blink response may reflect a failure to input or consolidate the second stimulus (T2) into working memory while T1 is being processed (Luck et al., 2000), thus supporting the utility of the P3 in documenting this effect. Consequently, the AB paradigm may help to elucidate the temporal nature of attention deficits in MDD and explain how select attentional processes are influenced by rumination levels. Although the P3 component in MDD has received scant attention using the RSVP paradigm, in general depressed patients show some reduction of the parietally maximal P3 component using a variety of oddball and go/no-go tasks (Bruder et al., 2012).

The present study used the flanker task and the RSVP paradigm to examine the relationship of rumination to response conflict and the temporal dynamics of attention in individuals diagnosed with MDD compared to healthy controls. Although recognizing the lack of agreement in the literature concerning the N2 potential in MDD, we hypothesized that individuals with a current diagnosis of depression would display relative deficits in conflict monitoring using a modified flanker task. We expected these deficits to manifest as reductions in N2 amplitude as well as impaired behavioral task performance outcomes. It was also hypothesized that individuals with MDD would exhibit selective deficits in attention (i.e., evidence a larger blink) during the rapid presentation of visual stimuli and larger T1- and T2-elicited P3 amplitudes. These findings would indicate that individuals with MDD have less efficient neural resource allocation, resulting in impaired ability to consolidate two temporally close items into working memory. It was predicted that individual differences in rumination would covary with both ERP and behavioral deficits in the performance of both tasks.

## Materials and Methods

### Participants

Individuals with MDD were solicited from a university counseling and psychiatric services clinic, where they were diagnosed prior to participation by a psychologist, psychiatrist, or primary care provider. Control participants were recruited through advertisements posted in numerous locations around the university campus and local community. Recruitment was for a mental and physical skill training program aimed at improving physical and psychological health; the data presented herein represent findings from the initial baseline assessment. Participants of all ethnic origins between 18 and 35 years of age were included in the study. Only right-handed participants with normal or corrected-to-normal vision were included in the current analyses. Participants in the MDD group were included if they met diagnostic (DSM-IV-TR) criteria for current MDD (American Psychiatric Association, 1994) and confirmed by the Mini-International Neuropsychiatric Interview (MINI; Sheehan et al., 1998). Exclusion criteria included a diagnosis of a bipolar spectrum disorder, schizophrenia spectrum disorder, self-injurious or suicidal behavior, or a history of neurological disorders or head injuries resulting in a loss of consciousness. All clinical interviews and suicide risk assessments were completed by trained graduate students working with the study PIs, and all were trained to competence by a licensed clinical psychologist (EAS), who supervised all study clinical assessments and is a member of the clinical psychology Ph.D. training program at Rutgers University. Prior to participating in clinical interviews, all clinical interviewers completed extensive clinical training regarding the clinical symptoms assessed with the MINI, including depression. This training included formal team meetings outlining the structure and content of the diagnostic interview, discussions on troubleshooting and differential diagnoses with the interview, extensive shadowing of Dr. Selby who completed all assessments during the first semester of data collection, and an examination interview that required rating the correct diagnoses for a case presenting pre-specified symptoms that all interviewers were required to pass. During the course of the study any concerns regarding diagnostic symptom endorsement or suicide risk concerns were discussed with the supervisor to ensure patient risk protection. The MINI is an appropriate tool for assigning threshold level psychiatric diagnoses in research settings and has been found to have strong diagnostic agreement with other clinical interviews such as the Structured Clinical Interview for DSM-IV Axis I Disorders (SCID; First et al., 1995), which are frequently used in clinical settings because they explore symptoms in more depth than is needed for a research study (Jones et al., 2005). Healthy comparison participants who did not meet criteria for MDD via the MINI were also recruited and included if they reported no previous or current history of neuropsychiatric disorders, neurological disorders, or head injuries. All eligible individuals were invited to visit the laboratory for a more extensive clinical interview and neurophysiological testing. The final sample consisted of 33 MDD participants and 36 healthy controls. MDD and control participants did not differ with respect to age, sex, ethnicity, or educational level, *p*s > 0.05. Six of the 33 MDD (18%) participants had a confirmed comorbid diagnosis at the time of participation [one with post-traumatic stress disorder, five with generalized anxiety disorder (GAD)] and three of these participants reported current antidepressant drug use. Additionally, two other participants were currently taking either antidepressant (*n* = 1) or ADHD (*n* = 1) medication. No medication use was reported among the control participants. As expected, MDD participants reported significantly increased levels of depressive symptoms assessed by the Beck Depression Inventory-II (BDI-II; Beck et al., 1961, 1996) and rumination as assessed by the Ruminative Responses Scale (RRS; Nolen-Hoeksema et al., 1999). **Table 1** shows participants’ demographic and behavioral data according to group status. The research protocol was approved by the university’s Institutional Review Board and written informed consent was obtained from all participants prior to participation.

**Table 1:** Demographic and clinical characteristics of participants by group status.

Characteristic	Control	MDD	Total
*n*	36	33	69
Age (years)	21.0 ± 3.1	20.7 ± 2.9	20.9 ± 2.9
Gender (male/female)	12/24	9/24	21/48
Height (cm)	166.6 ± 8.5	164.9 ± 7.9	165.8 ± 8.2
Weight (kg)	67.4 ± 15.0	66.3 ± 15.6	66.9 ± 15.2
BMI (kg/m^2^)	24.3 ± 5.1	24.3 ± 4.9	24.3 ± 5.0
BDI-II score	7.4 ± 4.8	23.9 ± 8.3^∗^	15.4 ± 10.7
RRS total	41.9 ± 10.6	59.1 ± 10.2^∗^	50.2 ± 13.5
Depression	21.8 ± 5.9	33.2 ± 6.6^∗^	27.3 ± 8.4
Brooding	9.7 ± 2.5	13.5 ± 3.6^∗^	11.5 ± 3.6
Reflection	10.4 ± 3.9	12.4 ± 3.2^∗^	11.4 ± 3.7

*Values equal mean ± SD. MDD, major depressive disorder; BMI, body mass index; BDI, Beck Depression Inventory; RRS, Ruminative Responses Scale. Asterisks indicate statistically significant unpaired student’s t-test between control and MDD participants, p < 0.05*.

### Procedures

Individuals meeting the initial study inclusion criteria were invited for a baseline testing session to complete a clinical interview and provide behavioral and neurophysiological data. After receiving a general description of the study and providing written informed consent, participants completed a set of questionnaires pertaining to their demographics, attitudes, mood, and health, including the BDI-II and RRS. Next, participants were fitted with a 64-channel Geodesic Sensor Net (Electrical Geodesics, Inc., Eugene, OR, USA) and seated ∼0.5 m directly in front of a 17” Dell computer monitor. After completing a 5-min rest period, participants completed the flanker and AB tasks in counterbalanced order. Following the neurocognitive assessments, participants completed a cardiovascular and physical fitness test battery that was part of the larger intervention study.

### MDD Diagnosis, Depression Symptoms, and Rumination Assessment

#### Mini Neuropsychiatric Diagnostic Interview

The MINI (manic/hypomanic episodes, obsessive-compulsive disorder, substance and alcohol use disorders) was used to confirm clinical diagnosis of MDD. The MINI is a brief structured interview that has been used extensively to aid in making diagnoses of Diagnostic and Statistical Manual of Mental Disorders, Fourth Edition (DSM-IV) and International Classification of Diseases-10 (ICD-10) psychiatric disorders. The reliability and validity of this instrument have been previously established (Lecrubier et al., 1997; Sheehan et al., 1997). The point biserial correlation coefficient of MDD diagnosis with BDI-II scores was 0.80, *p* < 0.001.

#### Depressive Symptoms

Participants completed the BDI-II (Beck et al., 1961), a 21-item, self-report inventory of the severity of current depressive symptoms. Higher total scores reflect greater subjectively perceived depressive symptomatology. The BDI-II in this sample demonstrated good internal consistency (α = 0.92).

#### Rumination

Participants completed the RRS (Nolen-Hoeksema et al., 1999), which includes 22 items describing thoughts and responses to depressed mood that are focused on the individual themselves, possible symptoms, and potential consequences/causes of the mood. They were asked to rate each item on a scale from 1 (almost never) to 4 (almost always). An example of one of the items is: “Analyze recent events to try to understand why you are depressed.” The RRS scale demonstrated appropriate internal consistency (α = 0.93).

### Cognitive Tasks

#### Eriksen Flanker

**FIGURE 1:**
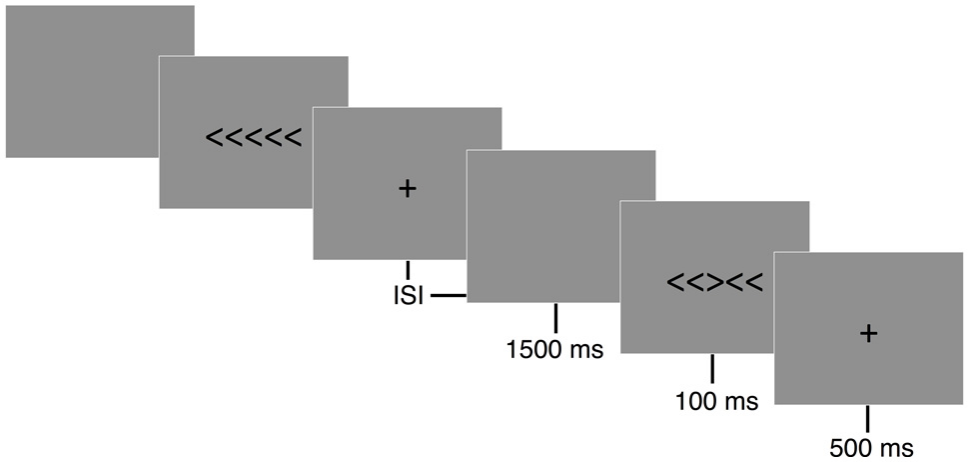
**Eriksen flanker task**. Following a 500 ms fixation cross (+), either congruent (< < < < <) or incongruent (< < > < <) stimuli were displayed focally for 100 ms on a computer screen. Participants were instructed to respond to the direction of a centrally positioned target arrow as quickly and accurately as possible. A button press corresponding to the direction of the centrally positioned arrow was recorded during a 1500 ms response window. A random inter-stimulus interval (ISI) of 1100, 1300, or 1500 ms occurred following the participant’s response to reduce expectancy effects.

A modified arrow version of the Eriksen flanker task (Eriksen and Eriksen, 1974) was presented with E-prime version 2.0 software (Psychology Software Tools, Inc., Pittsburgh, PA, USA). The flanker task is composed of two conditions, congruent and incongruent, during which participants are asked to press a button corresponding to the direction of a centrally positioned target arrow (see **Figure 1**). The congruent trials consisted of the target arrow being flanked by arrows facing the same direction (i.e., < < < < < or > > > > >), while incongruent trials involved the target arrow being flanked by arrows facing the opposing direction (i.e., < < > < < or > > < > >). A set of instructions preceded the first trial that explained which button press would be used to indicate the direction of the central or target arrow. Participants performed a button press with their left thumb when the target arrow, or third arrow from the left, pointed to the left (<) and a button press with their right thumb when the target arrow pointed to the right (>). Following task instructions, participants completed 20 practice trials. Performance feedback was provided on the computer screen and any remaining questions were answered during the practice trials to ensure participants sufficiently understood the task. Each trial began with a black screen containing a white fixation cross (+) in the middle of the screen for 500 ms, following which 7.6 cm tall stimuli were presented focally on a computer screen in white letters on a black background for 100 ms with a response window of 1500 ms and a variable inter-stimulus interval (ISI) of 1100, 1300, or 1500 ms. Participants were instructed to respond as quickly and accurately as possible for each trial. Two blocks of 110 trials were administered with equiprobable congruency and directionality of stimuli.

#### Attentional Blink

**FIGURE 2:**
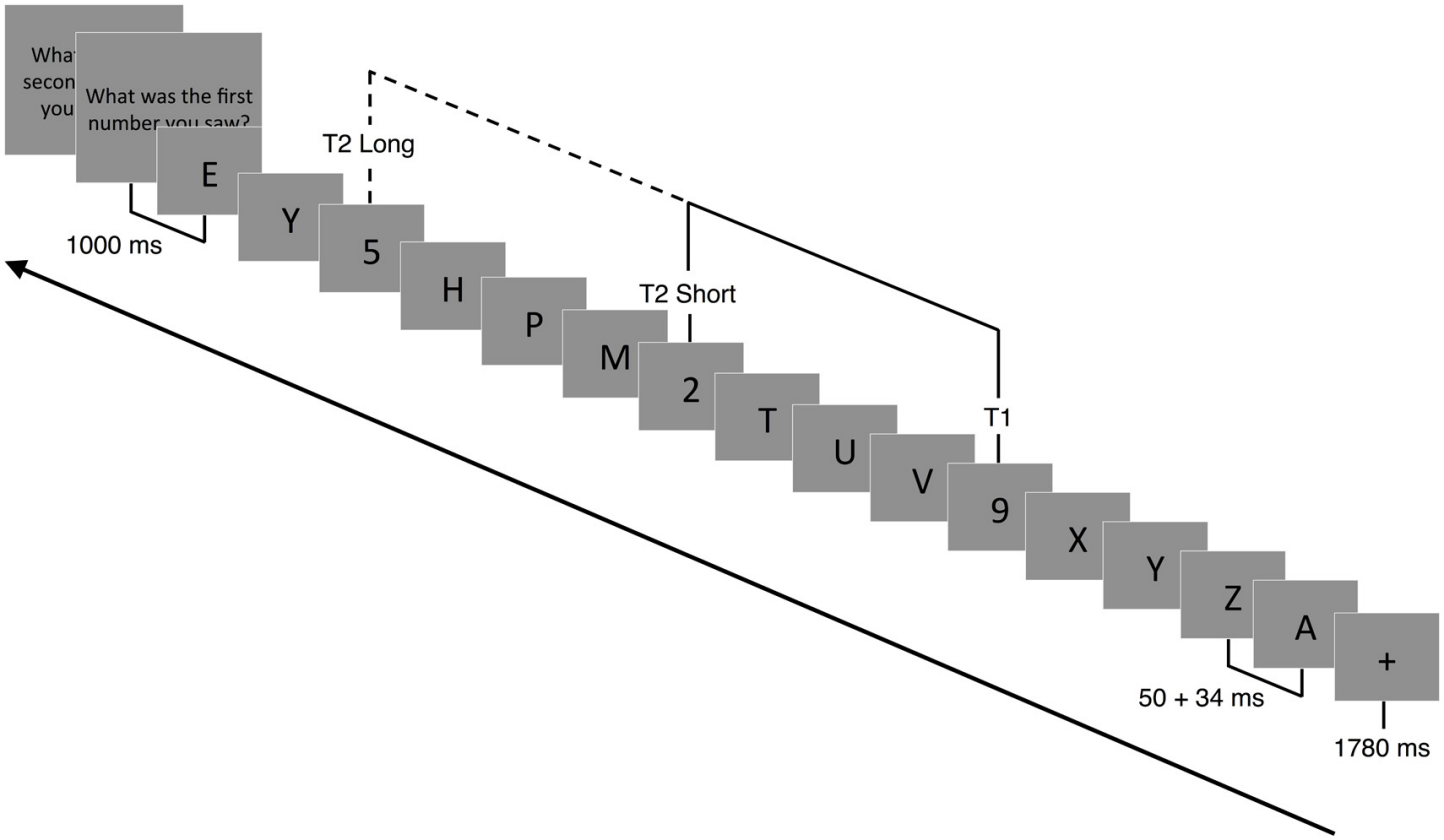
**Attentional blink paradigm**. Following a 1780 ms fixation cross (+), a RSVP of 15 or 19 capital letters (distractors) were displayed focally on a computer screen. Embedded within the RSVP were 1 (T1 present-T2 absent) or 2 (T1 present-T2 present) target numbers. Each slide was presented for 50 ms followed by a 34 ms blank slide. Participants had to detect T1 and T2 (if present) and report their response at the end of each trial. In T2 absent trials, T2 was replaced with a blank screen. The temporal lag between T1 and T2 could vary between 336 ms (short trials) and 672 ms (long trials).

A modified version of the AB task was adapted from Slagter et al. (2007) and used to assess competition between targets for limited attentional resources (Shapiro et al., 1997; Martens and Wyble, 2010). Stimuli were presented focally on a computer screen in white letters on a black background. Following the presentation of a 1780 ms fixation cross (+), a RSVP of 15 or 19 capital letters was presented. Participants were instructed to identify two target numbers (T1 and T2) embedded within the rapid visual stream of letters (distractors). Each stimulus was presented for 50 ms, followed by a 34-ms blank slide (**Figure 2**). For each trial, the letter (distractor) was randomly selected (without replacement) from the alphabet. Within each trial, one (single-target) or two (dual-target) letters were replaced with a randomly drawn (without replacement) number ranging from 2 to 9. In the case of single-target trials, the second target was replaced with a blank screen (T2-absent trial). The time between T1 and T2 (or the blank) was either 336 ms (short) or 672 ms (long). The shorter timeframe between targets (T2-present/short trials) has been shown to reliably produce a blink (misidentified T2) compared to the longer timeframe (T2-present, long trials). This occurs during peak competition for limited attentional resources (Slagter et al., 2007). T2 and the blank screen were presented in positions 3–5 from the end of the stream. Due to similarities, letters B, I, O, Q, and S and numbers 1 and 0 were omitted from the visual stream.

Prior to the task, participants were instructed that there could be one or two numbers (targets) in the letter stream. At 1000 ms after the stream was completed, participants were instructed to enter the numbers on a keyboard in the order they were presented. If participants were unable to identify T2, they were instructed to guess its identity. Participants entered zero if they were absolutely certain there was no second target presented. A new trial began 200 ms after the second number (or blank) was identified. In addition to verbal instructions, on-screen instructions and a 1-min practice block, with feedback, were provided prior to actual testing. Participants then performed two blocks of 112 trials each. There were a total of 116 T2-present/short trials, 36 T2-present/long trials, 36 T2-absent/short trials, and 36 T2-absent/long trials, which were randomly selected during each block.

### ERP Data

Electroencephalographic (EEG) activity was recorded using a 64-channel Geodesic Sensor Net system (Electrical Geodesics, Inc., Eugene, OR, USA) arranged according to the International 10–10 system (Chatrian et al., 1988). The electrooculogram (EOG) was recorded from electrodes located above and below each eye. Individual electrode impedances were kept below 50 kΩ in accordance with standard data collection procedures (Ferree et al., 2001). Although lower impedances are typically recommended (Luck, 2014), previous research has shown excellent EEG signals when data were collected with higher scalp impedance (Ferree et al., 2001; Keil et al., 2014), and similar values have been used in the study of ERP components in MDD (Clawson et al., 2013). Data were sampled at 250 Hz and collected with a 0.1–100 Hz bandpass hardware filter. Continuous data were recorded during each task condition referenced to the vertex electrode (Cz). Following collection, data were re-referenced (Bertrand et al., 1985; Tucker et al., 1994) to the mastoids and filtered with a 35 Hz low-pass filter. Data were visually inspected for eye-blinks, eye-movements, and bad channels before and after artifact rejection tools were applied to correct and remove ocular artifact using NetStation 4.0 (Electrical Geodesics, Inc., Eugene, OR, USA). Briefly, segments were marked “bad” if they contained (1) eye movements exceeding 55 μV, (2) eye blinks exceeding 140 μV, or (3) greater than or equal to 10 bad channels exceeding 200 μV. In each case, a moving average of 20 samples combined with threshold values were used. Using spherical spline interpolation, bad channels were then replaced from the remaining channels in “good” segments.

For the flanker task, epochs of individual trials were created from 100 ms pre- to 1000 ms post-stimulus presentation and baseline adjusted using the 100 ms pre-stimulus period. ERPs were constructed by averaging across congruent and incongruent trials separately for each participant. N2 amplitude was captured using a mean-amplitude approach. Consistent with previous research and based on visual inspection of waveforms (Krompinger and Simons, 2011; Keil et al., 2014; Luck, 2014), we used a window spanning 200-350 ms post-stimulus to evaluate the N2 (Folstein and Van Petten, 2008; Dickter and Bartholow, 2010). For the AB task, epochs of individual trials were created from 200 ms pre- to 2000 ms post-stimulus (relative to T1). All epochs were baseline corrected using the 200 ms pre-stimulus period. For short trials, the T1-elicited P3 components were defined within a 295–365 ms window post-stimulus, while the T2-elicited P3 components were defined within a 847–1151 ms window (Slagter et al., 2007). Additionally, on long trials, T1-elicited P3 components were averaged across a 295–651 ms window while the T2-elicited P3 components were defined within a 1147–1451 ms window (Slagter et al., 2007). We used a mean amplitude approach to isolate ERP components since this is viewed as a more unbiased estimation of ERP amplitude (Clayson et al., 2013; Luck, 2014). Artifact-free waveforms where the arrow directions were correctly identified (flanker) or both T1 and T2 targets were identified (AB) were subsequently grand averaged.

### Data Analysis

#### Behavioral Data

Only trials in which a response was attempted were considered. To reduce the potential effect of outliers, trials with RTs beyond the individual mean ± 3 SD for each trial type were excluded. Exploratory analyses using one-way analyses of variance (ANOVAs) revealed no significant effects of sex or ethnicity on any of the cognitive performance measures; therefore, these variables were not further considered. Behavioral performance data (i.e., response time and accuracy) from the flanker task were submitted to a 2 (Group: MDD patients, controls) × 2 (Task Congruency: Congruent, Incongruent) ANOVA with repeated measures. To assess performance on the AB task, average T1 and T2 accuracy data were submitted to a 2 (Group: MDD patients, controls) × 2 (Lag: Short, Long) ANOVA. Only trials in which T1 was identified correctly were examined. In order to determine the relationship between self-reported rumination and behavioral task performance, a bivariate Pearson correlation between rumination scores and accuracy and RT data for both cognitive tasks was performed.

#### ERP Data

In light of the frontocentral nature of the N2 component elicited by the flanker task, a mixed 2 (Group: MDD patients, controls) × 2 (Task Congruency: Congruent, Incongruent) × 3 (Site: Fz, FCz, Cz) ANOVA with repeated measures was conducted on mean N2 amplitudes. Less negative values were interpreted as reflecting less cognitive control in response to the stimulus. To investigate the relationship between rumination and N2, we performed bivariate Pearson correlations between self-reported rumination scores and N2 amplitudes for congruent and incongruent flanker trials. For the AB task, mixed 2 (Group: MDD patients, controls) × 2 (Lag: Short, Long) × 4 (Site: Fz, FCz, Cz, Pz) with repeated measures were conducted for P3 amplitudes corresponding to T1 and T2. Since previous research indicates that comorbid anxiety-related disorders and psychotropic medications may influence neural activation patterns (Gehring et al., 2000; de Bruijn et al., 2006; Hajcak et al., 2008) and psychomotor speed, we reanalyzed the behavioral and ERP outcomes while excluding those participants with comorbid diagnoses or current psychotropic medication use. We performed similar bivariate correlations for P3 amplitudes on T1 and T2 trials of the AB task. Partial eta squared ($\eta_{p}^{2}$) values are reported to demonstrate the magnitude of effect sizes (ESs) following ANOVAs, with 0.01–0.059 representing a small effect, 0.06–0.139 a medium effect, and >0.14 a large effect (Cohen, 1973). *Post hoc* comparisons were conducted using univariate ANOVAs and Bonferroni corrected *t*-tests. ESs were calculated for any pairwise comparisons by using Hedges’ g statistic (Hedges, 1981). A critical alpha level of *p* < 0.05 was adopted for all significance tests.

## Results

### Behavioral Data

**FIGURE 3:**
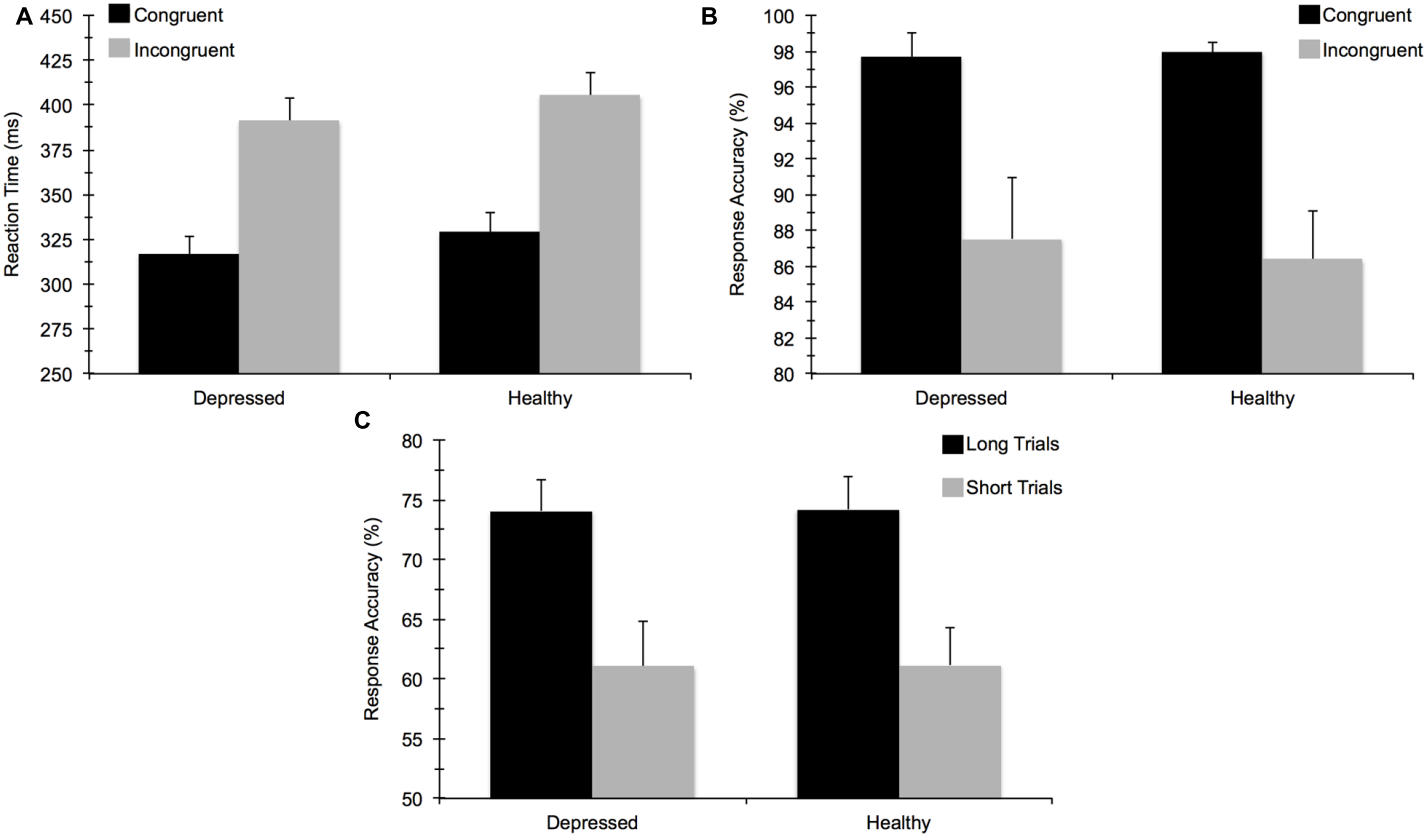
**Mean (±) SE behavioral task performance for: (A) reaction time (ms) on the flanker task, (B) response accuracy (%) on the flanker task, and (C) response accuracy on the attentional blink task**.

For accuracy on the flanker task, the mixed 2 (Group) × 2 (Task Congruency) ANOVA revealed a main effect of congruency, *F*(1,67) = 34.97, *p* < 0.001, $\eta_{p}^{2}$ = 0.34, indicating that participants performed worse on incongruent relative to congruent trials. No main effect of group or group by congruency interaction was observed, indicating that MDD and healthy controls did not differ in terms of overall accuracy. This test also confirmed that a comparable number of data points in each group were available for subsequent ERP analysis. The ANOVA for RT similarly showed a main effect of congruency, *F*(1,67) = 397.52, *p* < 0.001, $\eta_{p}^{2}$ = 0.86, due to faster response times for congruent versus incongruent trials. The main effect of group and the group × congruency interaction were not statistically significant. For the AB task, T2 accuracy was significantly higher on long trials (74%) than on short trials (61%), *F*(1,67) = 69.33, *p* < 0.001, $\eta_{p}^{2}$ = 0.51, confirming an AB effect whereby T2 was detected less frequently on the short trials. However, no significant group main effect or interaction with group was observed. In sum, MDD and healthy control participants did not differ in terms of behavioral task performance for either task (see **Figure 3**). These findings remained consistent when we reanalyzed the data to account for comorbid diagnoses or current medication use. No significant correlations emerged between self-reported rumination and behavioral performance measures for either cognitive task.

### ERP Data

**FIGURE 4:**
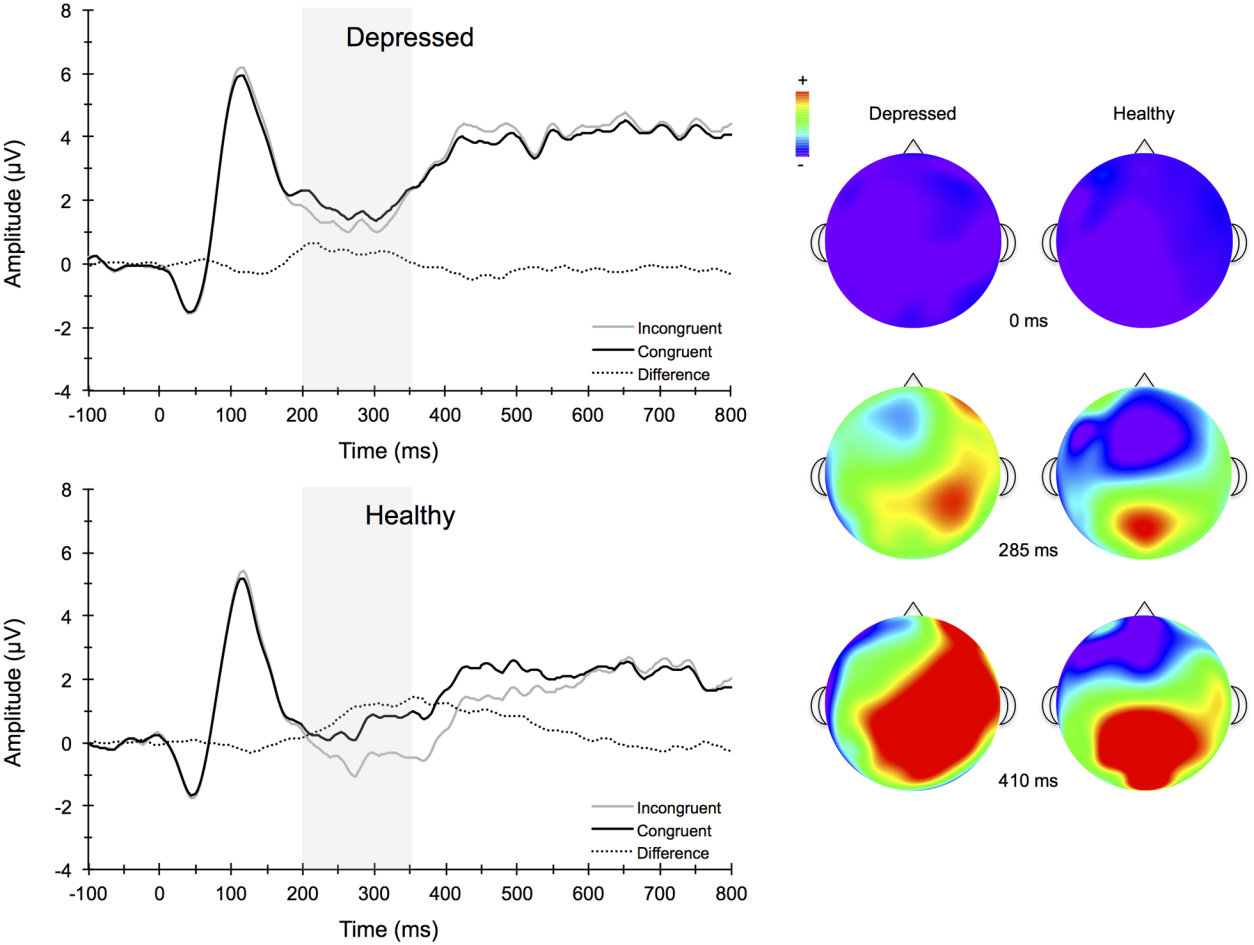
**Stimulus-locked grand average ERP waveforms for congruent (top left) and incongruent (bottom left) flanker conditions averaged across frontocentral midline electrode sites Fz, FCz, and Cz**. Topographic scalp maps (right) collapsed across congruency for depressed and healthy participants.

**FIGURE 5:**
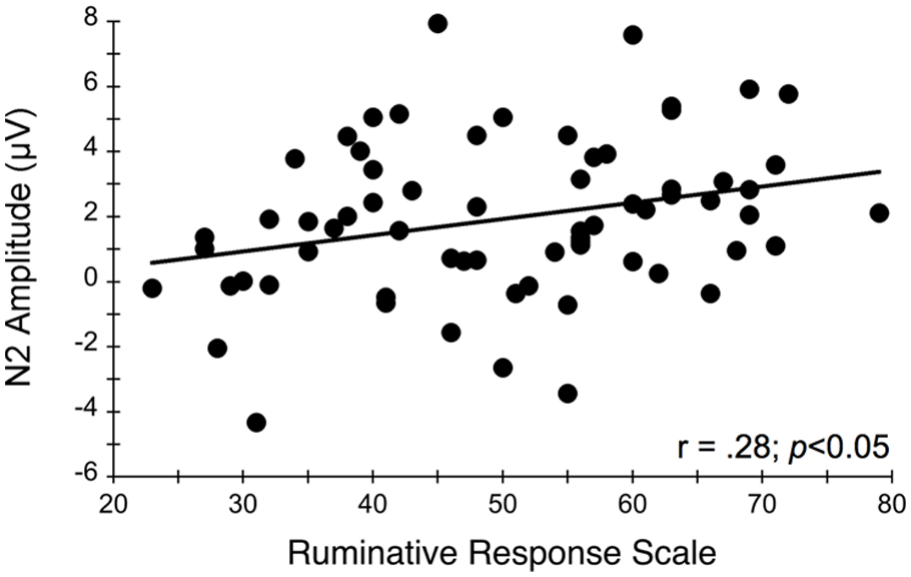
**Relationship between N2 amplitude averaged across frontocentral midline electrode sites Fz, FCz, and Cz and self-reported rumination levels during the flanker task**. More positive N2 amplitudes are interpreted as an index of impaired conflict monitoring.

**Figure 4** illustrates the grand averaged ERP waveforms for each group at the three frontocentral midline electrode sites (Fz, FCz, Cz) to congruent and incongruent flanker task stimuli. The total number of flanker trials used for ERP analysis did not differ by group or condition. ERPs for MDD participants included a total of 88 ± 16 trials for the incongruent condition and 88 ± 15 trials for the congruent condition and ERPs for control participants included a total of 93 ± 12 trials for the incongruent condition and 92 ± 13 trials for the congruent condition (mean ± SD). For the N2 component, a Group (MDD, controls) × Task Congruency (Congruent, Incongruent) × Site (Fz, FCz, Cz) ANOVA revealed a main effect of Group, *F*(1,67) = 6.28, *p* = 0.015, $\eta_{p}^{2}$ = 0.09, Task Congruency, *F*(1,67) = 16.48, *p* < 0.001, $\eta_{p}^{2}$ = 0.20, and Site, *F*(2,66) = 27.63, *p* < 0.001, $\eta_{p}^{2}$ = 0.46. *Post hoc* Bonferroni corrected *t*-tests of the Group main effect indicated that healthy controls demonstrated overall more negative activity (0.348 μV) in the N2 latency time window than the MDD group (-1.483 μV), *p* = 0.015. The Congruency main effect showed that N2 displayed a larger response (more negative amplitude) for incongruent (-0.967 μV) relative to congruent (-0.167 μV) flanker trials, *p* < 0.001. Decomposition of the Site main effect revealed significantly more negative amplitudes for Fz (-1.442 μV) and FCz (-1.036 μV) sites relative to Cz (0.777 μV), *p*s < 0.05. The Group and Task Congruency main effects were superseded by a significant Group × Task Congruency interaction, *F*(1,67) = 4.20, *p* < 0.05, $\eta_{p}^{2}$ = 0.06. This interaction revealed a larger flanker N2 effect (i.e., larger N2 for incongruent versus congruent task conditions) for healthy participants (1.204 μV), ES = 0.38, *p* < 0.001, compared to MDD participants (0.396 μV), ES = 0.13, *p* = 0.131. Significant main effects of Group, Congruency, and the Group × Task Congruency interaction remained significant when accounting for comorbid diagnosis and current medication use (*F*s > 3.7, *p*s < 0.05), suggesting that these differences did not alter the overall pattern of findings. Importantly, self-reported rumination was also significantly correlated with N2 amplitude, *r* = 0.28, *p* = 0.02 (see **Figure 5**). This positive correlation indicates that as rumination scores increased, N2 amplitude became more positive (i.e., less negative N2 amplitude reflects reduced cognitive control).

**FIGURE 6:**
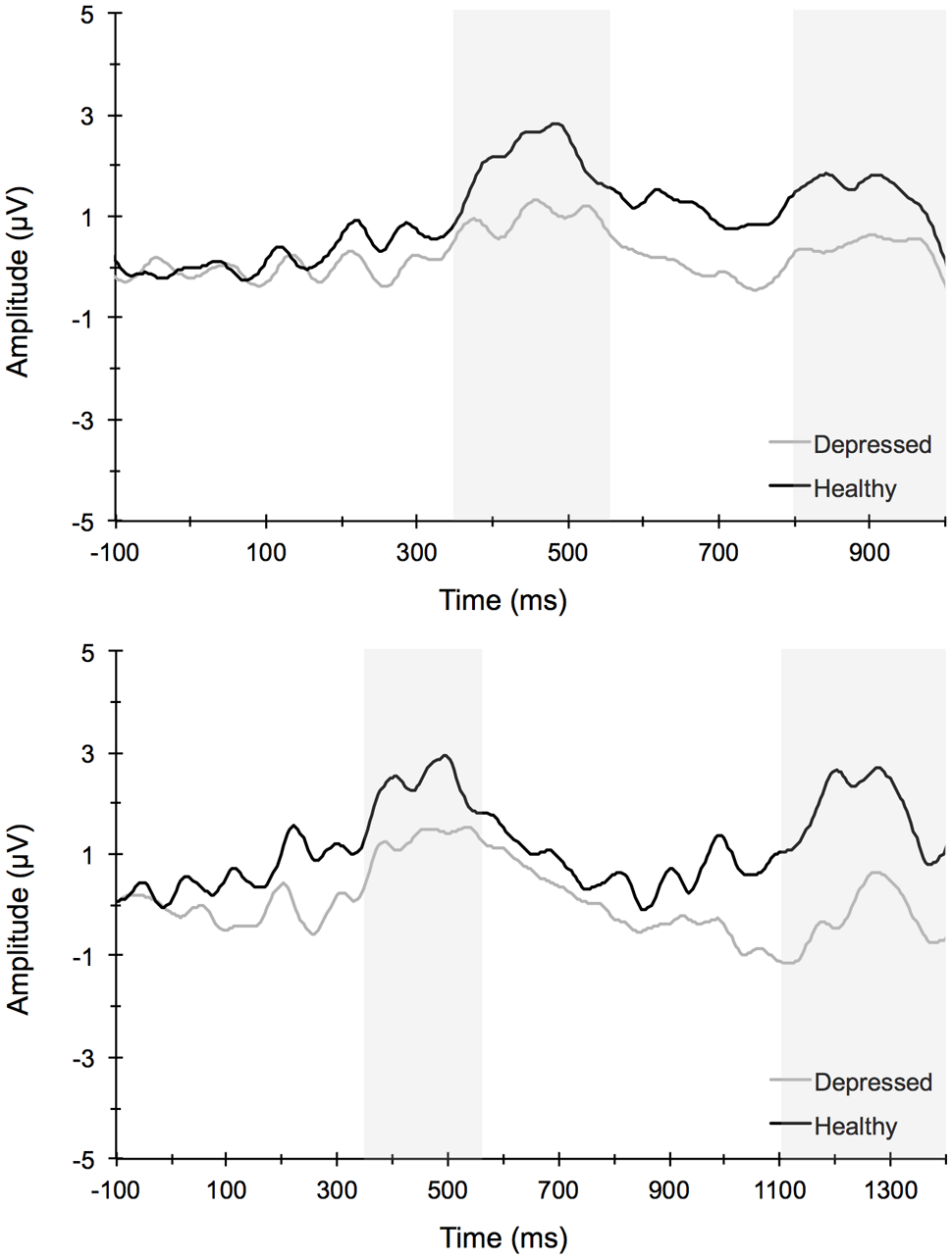
**Stimulus-locked grand average waveforms for short (top) and long (long) attentional blink trials averaged across centroparietal midline electrode sites Cz, CPz, and Pz**.

**Figure 6** illustrates the grand averaged ERP waveforms for each group averaged across the Cz, CPz, and Pz midline electrode sites to short and long trials of the AB task. The total number of AB trials used for ERP analysis did not differ by group or condition. ERPs for MDD participants included a total of 80 ± 21 trials for the short lag condition and 23 ± 5 trials for the long lag condition. Similarly, ERPs for control participants included a total of 80 ± 26 trials for the short lag condition and 22 ± 4 trials for the long lag condition (mean ± SD). For the AB task, the omnibus analysis for P3 amplitude to T1 yielded a significant main effect for Lag, *F*(1,67) = 11.25, *p* < 0.001, $\eta_{p}^{2}$ = 0.14, and Site, *F*(3,65) = 21.79, *p* < 0.001, $\eta_{p}^{2}$ = 0.50, with *post hoc* analyses for Lag revealing significantly larger T1-elicited P3 amplitudes in long trials compared to short trials and for Site revealing a central-parietal distribution, with the parietal and central sites showing significantly larger amplitudes than the frontal and frontocentral sites, *p*s < 0.01. No group level main effects or interactions were evident for T1-elicited P3 amplitude. The 3-way mixed model ANOVA for P3 amplitude elicited by T2 similarly revealed a significant main effect for Lag, *F*(1,67) = 19.66, *p* < 0.001, $\eta_{p}^{2}$ = 0.23, and Site, *F*(3,65) = 4.75, *p* < 0.01, $\eta_{p}^{2}$ = 0.18, with *post hoc* analyses revealing larger T2-elicited P3 amplitudes on long trials compared to short trials and for Site revealing larger P3 amplitudes over central and parietal electrode sites compared to frontal and frontocentral sites. No significant main effects or interactions by Group were found. Self-reported rumination levels were not associated with T1- or T2-elicited P3 amplitudes for short or long trials (*p* > 0.05).

## Discussion

Individuals who suffer from MDD often experience deficits in learning, memory, selective attention, and cognitive control (Veiel, 1997; Zakzanis et al., 1998; Austin et al., 2001; Hammar and Ardal, 2009). These various processes allow one to initiate actions, evaluate risks, make decisions, plan for the future, inhibit habitual or prepotent responses, and resist temptations. During depressive episodes, individuals often ruminate about the past, which further interferes with cognitive control processes and the ability to inhibit unwanted thought patterns. In this study, we examined conflict monitoring using a modified flanker task and the ability to process two temporally close stimuli using a RSVP paradigm in depressed individuals and healthy controls, as well as the relationship of these cognitive processes to rumination. We examined how these behavioral and psychological measures related to neural indices of cognitive control and attention as evident in ERPs elicited by the flanker and AB tasks. The present findings indicate that although behavioral task performance was comparable between the two groups, there were differences between the groups’ respective ERP responses to environmental stimuli. ERPs may be more sensitive to subtle cognitive dysfunction in MDD and provide some insight into the underlying mechanisms involved. Consistent with our hypotheses, the amplitude of the N2 was significantly reduced in depressed participants when compared to non-depressed participants. These neuronal responses were particularly observed in response to the incongruent flanker task condition (i.e., the task condition requiring greater amounts of conflict monitoring) and were maximal at frontocentral recording sites. Moreover, correlation analyses indicated that participants who reported greater rumination levels also expressed significantly smaller N2 amplitudes. Contrary to expectations, behavioral performance and P3 amplitudes for the AB task were similar among depressed and non-depressed individuals, suggesting preserved temporal dynamics of attentional processes and neural resource allocation during the AB task in MDD. The implications of these findings are discussed below.

First, similar to several previous studies (Krompinger and Simons, 2011; Clawson et al., 2013) we failed to observe any differences in behavioral performance measures between individuals with MDD and typical controls. Moreover, no significant correlations were found between self-reported rumination and accuracy or reaction time measures for either cognitive task. In contrast, although Holmes and Pizzagalli (2008) reported no difference in accuracy, they found significantly longer response times for MDD patients relative to controls for the more challenging incongruent Stroop task trials. The participants in this latter study were older than those used in our study or in these previous studies of cognitive control deficits in MDD (Krompinger and Simons, 2011; Clawson et al., 2013). Many of the participants in our study were also university students. It is possible that select neurocognitive deficits in MDD are not observable using behavioral performance measures in such a young, otherwise high functioning population. One advantage of ERPs over behavioral measures is that they can provide information regarding the covert subset of neural processes that occur between stimulus engagement and motor response execution. Therefore, they may be more suitable to detecting any underlying impairment in conflict monitoring and attentional processes that may be present yet not observable through other measures. Future studies aimed at investigating the temporal sequence of cognitive deficits and MDD as well as any developmental variations in these neurocognitive processes are warranted.

Depressed individuals displayed lower N2 ERP amplitudes during the flanker task, a task requiring variable amounts of cognitive control. The ERP data suggested impairment in early conflict processing stages of information processing. This suggests that the MDD group recruited less cognitive control during task performance compared to healthy controls. Previous studies examining the N2 component in MDD have resulted in mixed findings, and several recent studies have not found between group (MDD versus controls) differences in N2 amplitude (Ruchsow et al., 2008; Clawson et al., 2013). Clawson et al. (2013) examined N2 amplitudes in the context of conflict adaptation, wherein previous trial congruency influences current-trial performance through available cognitive control resources. Although no group differences were found, depressive symptoms assessed through the BDI-II were significantly correlated with N2 conflict adaptation scores. The authors noted that the relationship between symptoms of depression and reduced conflict adaptation processes may be dimensional rather than categorical in nature, and that individuals who experience levels of depression that fail to meet diagnostic thresholds may still evidence cognitive dysfunction, including those processes involved in conflict adaptation. In contrast, Mao et al. (2005) assessed an N2 component (labeled the N270 in their study) during a visual S1–S2 mismatch paradigm and found smaller N270 amplitude among the depressed patients compared to controls at frontal and parietal electrode sites. This finding was interpreted as evidence of impaired conflict processing, involving ACC and lateral PFC regions. Differences in the tasks used, clinical characteristics of the participants, and precise timing of the component amplitudes across studies may have resulted in the mixed findings in the literature. Future studies should incorporate tasks that are believed to be sensitive to both early and later stages of cognitive control processes to elucidate the precise temporal nature of dysfunction in MDD. Moreover, it will be important for future research to examine this relationship across a wider domain of depression severity to determine whether this relationship is stronger for more severely depressed individuals versus those with mild to moderate depression.

Several previous studies have also studied other ERP components associated with cognitive control processes in depressed patients using both emotional (Mitterschiffthaler et al., 2007; McNeely et al., 2008) and non-emotional (Holmes and Pizzagalli, 2008; Katz et al., 2010; Krompinger and Simons, 2011; Clawson et al., 2013) tasks. Using a Stroop task, Holmes and Pizzagalli (2008) found that individuals with MDD showed larger Stroop interference effects and reduced N2 and N450 amplitudes. The N450 component belongs to the conflict-monitoring family of ERPs and is believed to represent similar cognitive control processes related to conflict monitoring (i.e., N2), although it may not be as sensitive to conflict adaptation processes (Larson et al., 2014). Moreover, source localization analyses revealed reduced activation within dorsal ACC and left dorsolateral PFC regions 620 ms after stimulus presentation among MDD participants, and the reduced activation resulted primarily in response to incongruent trials (Holmes and Pizzagalli, 2008). Krompinger and Simons (2011) similarly found overall reduced Stroop N450 amplitudes in undergraduates with high BDI scores relative to those with lower BDI scores; however, a large Stroop congruency effect was found for the N450 for the high but not for the low depressive group, amid comparable behavioral task performance scores. This hyperactivation was also related to rumination and suggests that trait ruminators might over engage cognitive control processes, including the affective subdivisions of the ACC, in the process of performing at normative levels. It is also possible that depressed individuals have less efficient neuronal resources due to other psychological processes that occur during performance of the task, which may happen with trait ruminators. To examine this possibility, we assessed the correlation between individual differences in rumination and N2 amplitudes elicited by the flanker stimuli. We found that N2 amplitudes were significantly related to self-reported rumination levels, such that higher rumination scores were related to lower N2 amplitude. Thus, rumination appears to be associated with less efficient conflict monitoring resources used to attend to stimuli in the environment that may be unexpected and require the upregulation of cognitive control. This diminished cognitive control at the neurological level may partially explain why some depressed individuals have difficulty disengaging from negative environmental experiences and a diminished ability to reappraise situations to find positive perspectives about the situation (Troy et al., 2010). These findings are consistent with other studies, which report that clinically depressed individuals have difficulty inhibiting the emotional effects of negative content (Goeleven et al., 2006; Lau et al., 2007), even after the depression has remitted (Joormann, 2004; Joormann and Gotlib, 2007).

Deficits of cognitive control in depression could be caused by a number of neurobiological factors such as epigenetic influences, neurovascular changes, stress exposure, or environmental and social influences, among others. These influences likely interact to confer risk for MDD in particular and psychopathology more broadly. A recent meta-analysis of 193 structural neuroimaging studies across six diverse psychiatric diagnostic groups (schizophrenia, bipolar disorder, depression, addiction, obsessive-compulsive disorder, and anxiety) found gray matter loss in three specific brain regions across diagnostic categories: the dorsal anterior cingulate, right insula, and left insula (Goodkind et al., 2015). This finding was important not only in demonstrating a possible shared disrupted neural circuitry across diagnoses, but highlights the potential importance of executive functioning or cognitive control in these conditions. Others have previously noted important brain regions in depression (prefrontal cortex, hippocampus, and amygdala; Davidson et al., 2002), and, importantly, these structures also emerged as critical to depression in the meta-analysis (Goodkind et al., 2015). Depression may result in underlying neurobiological changes that in turn cause impairment in cognitive control (Snyder, 2013). The causal pathway may be explained by underlying impairments in cognitive control contributing to risk for or relapse of depressive episodes, possibly by contributing to an inability to inhibit feelings of frustration, helplessness, and low self-worth (Hammar and Ardal, 2009). As mentioned previously, there is a need for longitudinal studies to examine the directional links between cognitive control processes and depression at both a behavioral and neurobiological level.

A number of different attentional processes have been studied in relation to mood disorders and MDD (Hammar and Ardal, 2009; Lyche et al., 2011). Previous studies have indicated that the temporal dynamics of attentional processes can be influenced by demographic, lifestyle, or health factors, as reflected by the size of the AB as well as P3 amplitude (Martens et al., 2006; Slagter et al., 2007; Wu and Hillman, 2013), but how temporal dynamics of attention are affected by MDD is not well-understood. In the present study, we observed no group differences in attentional resources devoted to the AB task in participants with MDD. Specifically, no significant differences in the magnitude of the AB or P3 amplitude were observed between depressed and healthy groups. It is possible that individuals with depression can maintain attention through basic attentional tasks (e.g., simple organization tasks), but have more difficulty as the tasks require greater response monitoring or conflict (e.g., cognitive evaluation, aspects of social engagement). It is important in future research to examine the temporal sequence between MDD and conflict monitoring aspects of cognitive control. Our results suggest the possibility that an initial target of treatment in MDD should be the reduction of rumination.

While these data suggest impaired conflict monitoring in MDD, several limitations should be noted. First, the current sample size was relatively small, which may have limited power in detecting group differences in subtle attentional processes. Second, although group differences emerged in ERPs that were elicited by the flanker task, we did not observe statistically significant differences in behavioral task performance between depressed and healthy participants. We also did not find any significant correlations between N2 and P3 amplitudes and task performance measures. Previous studies have shown a relation between N2 amplitudes and reaction time (Yeung et al., 2004; Yeung and Nieuwenhuis, 2009; Clayson and Larson, 2011), suggesting that the degree of conflict as measured my N2 amplitude is reflected in impaired behavioral performance. It is possible that our version of the flanker task was not demanding enough to detect behavioral differences by depression status. Another possible explanation may be that our behavioral tasks did not involve a negative valence component, which may have activated depression symptomology more than the neutral behavioral stimuli used in this study. Further, it is possible that the MDD sample, consisting of enrolled undergraduate students, was functioning at a sufficiently high cognitive level to overcome potential neurocognitive limitations on the tasks employed. As mentioned previously, the precise temporal resolution of ERPs provides a more sensitive approach for detecting cognitive deficits and their underlying neurophysiological mechanisms. Although we did not source-localize our ERP components, the nature of the cognitive tasks and the ERP findings nonetheless allow us to draw comparisons to those observed in other ERP studies examining cognitive control processes.

In spite of these limitations, the present findings suggest a potential link between conflict monitoring processes and rumination in MDD. This study represents an initial step in developing a more comprehensive understanding of depression by integrating neural and cognitive models of MDD. The ERP data combined with previous source localization and fMRI studies suggest dysregulation within anterior cingulate and prefrontal brain regions in MDD (Holmes and Pizzagalli, 2008; Pizzagalli, 2011). Given these and related data, it is important to develop clinical interventions which increase the neuronal response that underlies conflict monitoring processes and reduce the maladaptive levels of rumination that often are observed in individuals with MDD.

## Conflict of Interest Statement

The authors declare that the research was conducted in the absence of any commercial or financial relationships that could be construed as a potential conflict of interest.
